# Empirical Evidence for Extended Cognitive Systems

**DOI:** 10.1111/cogs.13060

**Published:** 2021-11-11

**Authors:** Luis H. Favela, Mary Jean Amon, Lorena Lobo, Anthony Chemero

**Affiliations:** ^1^ Department of Philosophy University of Central Florida; ^2^ Cognitive Sciences Program University of Central Florida; ^3^ School of Modeling, Simulation, and Training University of Central Florida; ^4^ Departamento de Psicología Universidad a Distancia de Madrid; ^5^ Department of Philosophy University of Cincinnati; ^6^ Department of Psychology University of Cincinnati

**Keywords:** Enactive Torch, Extended cognition, Nonlinear dynamics, Recurrence, Self‐similarity, Sensory substitution

## Abstract

We present an empirically supported theoretical and methodological framework for quantifying the system‐level properties of person‐plus‐tool interactions in order to answer the question: “Are person‐plus‐tool‐systems extended cognitive systems?” Nineteen participants provided perceptual judgments regarding their ability to pass through apertures of various widths while using visual information, blindfolded wielding a rod, or blindfolded wielding an Enactive Torch—a vibrotactile sensory‐substitution device for detecting distance. Monofractal, multifractal, and recurrence quantification analyses were conducted to assess features of person‐plus‐tool movement dynamics. Trials where people utilized the rod or Enactive Torch demonstrated stable “self‐similarity,” or indices of healthy and adaptive single systems, regardless of aperture width, trial order, features of the participants’ judgments, and participant characteristics. Enactive Torch trials exhibited a somewhat greater range of dynamic fluctuations than the rod trials, as well as less movement recurrence, suggesting that the Enactive Torch allowed for more exploratory movements. Findings provide support for the notion that person‐plus‐tool systems can be classified as extended cognitive systems and a framework for quantifying system‐level properties of these systems. Implications concerning future research on extended cognition are discussed.

## 1 Introduction

Two commitments are commonly accepted as central to cognitive science: First, cognition is defined in terms of computations and representations. Second, cognition is localized in brains (e.g., Von Eckardt, 1995; Thagard, 2020). With that being said, alternative research programs that challenge one or both of these commitments have been fruitfully practiced contemporaneously. In regard to the first, it has been argued that “cognition” is not best understood in terms of either computations or representations but instead in terms of dynamical systems theory (e.g., van Gelder, 1998). In regard to the second, instead of being localized in brains, it has been argued that cognition is *embodied* in both the nervous system and nonneural morphology (e.g., Varela, Thompson, & Rosch, 1991/2016); *distributed* across brains, bodies, and features of the environment (e.g., Hutchins, 1995); and/or *situated* in the current biological and environmental context (e.g., Robbins & Aydede, 2009).

In this vein, cognition is *extended* when, during a cognitive task (e.g., decision making, goal‐directed behavior, etc.), the cognitive system incorporates features of the world whose spatial locations are beyond what is typically understood to be the boundaries of an organism, such as scales, shell, or skin (Favela & Chemero, 2016). From this approach, cognition is not just a neuronal phenomenon but can involve features of the body (e.g., cardiovascular system and limbs) and the world (e.g., other organisms and nonbiological tools like smartphones and wheelchairs; for review of additional examples, see Clark, 2008; Smart, 2017; Wagman & Chemero, 2014). This understanding of cognition falls along a spectrum. At the weaker end is the view that nonneuronal elements play significant *causal roles* in cognitive processes. In this way, cognition remains primarily neuronal but has strong causal relationships (e.g., coupling) with, for example, a chalkboard, pencil and notebook, or smartphone (Clark, 2008). At the stronger end of the spectrum is the view that nonneuronal elements play *constitutive roles* in cognitive processes. In this way, some cognitive processes are *made of* nonneuronal elements, for example, the information in a smartphone's hard drive could be a part of the user's overall memory system in a manner that is functionally equivalent to the information in their brain.

It is worth noting that there is disagreement as to whether or not particular cases count as extended cognition or if they are instead cases of distributed, embodied, or situated cognition (cf. Amon & Favela, 2019; Robbins & Aydede, 2009). On the one hand, these distinctions are verbal disputes that hinge on disagreements about the definitions of concepts.^1^Although we acknowledge that such definitional distinctions may have a significant impact on certain projects, our present aim is not to provide thorough arguments for the necessary and sufficient conditions of instances of extended cognition. On the other hand, such disagreements are indicative of a major weakness undermining the ability to make progress on these issues. Specifically, most debates concerning whether something is a case of extended cognition hinge on arguments motivated by appeal to intuitions and thought experiments (e.g., Adams & Aizawa, 2008; Clark & Chalmers, 1998). With few exceptions, do such arguments appeal to experimental data to support their claims (for review, see Wagman & Chemero, 2014)? With that said, even the literature that does appeal to experimentation is limited in that it attempts to leverage data that did not test hypotheses explicitly about extended cognition per se (e.g., Wagman & Hajnal, 2016; for the rare exception, see Dotov, Nie, & Chemero, 2010).^2^

The current work contributes to the empirical literature on extended cognition by presenting results from an experiment that centered on a research question explicitly about extended cognition: “Are person‐plus‐tool‐systems extended cognitive systems?” We approached this question by analyzing the movement dynamics of participants engaged with tools during a task‐centered on affordance judgments. *Affordances* are perceivable opportunities for behavior (Gibson, 1979/1986). The affordance “graspable,” for example, is perceived when a monkey sees a piece of fruit it can hold in its hand; if a piece of fruit is too large, then it would not *afford* grasping. Affordance‐based tasks provide an experimental methodology for evaluating various perceptual modalities (e.g., audition) and conditions (e.g., stepping on stairs of varying height; e.g., Warren, 1984). Whereas it is common for experimental psychology experiments to evaluate participants’ task performance in terms of absolute units of measurement (e.g., Euclidean distance), experiments centered on affordances do so in terms of action‐scaled or body‐scaled metrics. In an affordance‐based task, a participant's performance is assessed in terms of *action‐scaled* units, or a metric based on their action capabilities, such as how high they can step. Performance can also be evaluated in *body‐scaled* units, or a metric based on their body's measurements, such as their leg length. Employing these metrics allows an experimenter to contextualize task performance relative to participants’ action capabilities and body measurements. As such, the question for an affordance‐based task is not, “Can participants step on a stair height of X centimeters,” but rather “What action‐scaled and body‐scaled metrics differentiate stairs that are step‐on‐able from not‐step‐on‐able?” For example, the critical point exists at which an aperture affords passing through or not based on a participant's shoulder width. This critical point is described in terms of an *A*/*S* ratio, or aperture‐to‐shoulder ratio (Warren & Whang, 1987). Thus, a well‐functioning perceptual system is sensitive to its own action‐ and body‐scaled properties while perceiving what is afforded and what is not. A vast number of experiments have been conducted on the perception of affordances via biological senses, such as audition and vision (for review, see Turvey, 2019). There have also been a number of affordance‐based experiments involving nonbiological tools, such as manually wielding a wooden rod to judge the crossability of gaps (Burton, 1992) and a head‐mounted wooden dowel to judge a surface's standability (Wagman & Hajnal, 2016). Such examples demonstrate that affordances and their means of assessment (e.g., action‐scaled units) provide a rich basis for assessing perceptual capabilities via biological and nonbiological means in a wide range of experimental conditions.

Here, participants utilized vision or haptic sensory‐substitution devices (SSDs) to support perceptual judgments of affordances involving the task of passing through apertures (cf. de Paz, Travieso, Ibáñez‐Gijón, Bravo, Lobo, & Jacobs, 2019). Participants made perceptual judgments about whether they could walk through apertures of various widths either using unrestricted vision or blindfolded while wielding one of two haptic SSDs: a wooden rod or the Enactive Torch (ET; Froese, McGann, Bigge, Spiers, & Seth, 2012), a device that provides its user with distance information in the form of vibrotactile stimulation to the wrist.^3^ Movements were analyzed via dynamical systems theory methods in order to assess for the presence or absence of self‐similar dynamic structures. An example of self‐similarity is fractal geometry (Mandelbrot, 1977/1983) or patterns that exhibit the same structure at various spatial or temporal scales. Abstract fractals—for example, Cantor set, Koch snowflakes, and Mandelbrot set—exhibit perfect self‐similarity; that is, the shape exhibited at one spatial scale is exactly repeated at other scales. Fractal patterns in nature—for example, broccoli, coastlines, and lung bronchial tubes—are not perfectly self‐similar but exhibit statistical self‐similarity; that is, elements of a phenomenon are repeated at various scales.

One reason self‐similarity that has become a topic of interest for cognitive scientists is the fact that such patterns seem to be associated with a variety of cognitive phenomena. Self‐similarity is exhibited by, for example, the spatial organization of physiology associated with cognition (e.g., neuronal dendritic branching; Di Ieva, 2016) and, especially, the temporal activity of such physiology (e.g., Rubinov, Sporns, Thivierge, & Breakspear, 2011), as well as cognitive behavior itself (e.g., Holden, Van Orden, & Turvey, 2009). Another reason for the interest in self‐similarity is that such structures are associated with healthy systems (Massobrio, de Arcangelis, Pasquale, Jensen, & Plenz, 2015). “Healthy” has various meanings here, such as efficient and maximum information processing (Beggs, 2019), organismic homeostasis (Hausdorff, Peng, Ladin, Wei, & Goldberger, 1995; Peng et al., 1995), and system adaptability (Torre, Vergotte, Viel, Perrey, & Dupeyron, 2019) and integrity (Goldberger et al., 2002). Self‐similarity is a common feature of both natural and artificial systems (e.g., cardiovascular system, central nervous system, protein‐protein interaction networks, social networks, and World Wide Web; Di Ieva, 2016; Gallos, Song, & Makse, 2007; Goldberger et al., 2002; Hausdorff et al., 1995; Peng et al., 1995). Additionally, fractals are widely implicated as a diagnostic tool, where reductions in the fractal structure are associated with diseases—for example, deterioration of the spatial fractal structure of white matter is associated with neurodegenerative disease (Zhang & Yue, 2016) and temporal fractal structure of heartbeats as an indicator of heart disease (Peng et al., 1995). Due to this fact, assessing the degree of self‐similarity that a phenomenon exhibits—spatial or temporal—can serve *part* of the case that the phenomenon is a single, well‐functioning system. Accordingly, a collection of elements can be understood as a single “system” if it exhibits self‐similarity (e.g., fractals, power laws, scale invariance, etc.) via its organization and/or dynamics, which indicates that it is maximizing information processing, and maintains homeostasis while being balanced between adaptability and stability. Following from this line of thought, well‐functioning perceptual systems ought to exhibit various forms of self‐similar dynamics while accurately perceiving, such as while perceiving affordances (e.g., Hajnal, Clark, Doyon, & Kelty‐Stephen, 2018).

Note that we do not claim that one form of self‐similarity (e.g., monofractal) will alone demonstrate that a collection of elements is a single system. We build our research question on the following commitments: First, there is a compelling empirical literature demonstrating that single systems exhibit self‐similarity in their dynamics (e.g., heartbeats; see references above). Second, there is also compelling empirical literature demonstrating that single systems can exhibit self‐similarity in their spatial organizational structures (e.g., neuronal networks; see references above). Third, self‐similarity has been demonstrated by properly functioning single systems, where “properly” refers to healthy, highly efficient information processing, and/or accurate task completion. Fourth, various forms of self‐similarity are increasingly being implicated as a fundamental feature of a range of cognitive processes (for a small sample see Amazeen, 2018; Amon, Pavlov, & Holden, 2018; Chemero, 2009; Favela, 2020; Gilden, 2001; Gilden, Thornton, & Mallon, 1995; King, 1991; Van Orden, Holden, & Turvey, 2005; Van Orden, Hollis, & Wallot, 2012; Ward, 2002; Werner, 2010).

Addressing our overarching research question—“Are person‐plus‐tool‐systems extended cognitive systems?”—required us to address the following related questions: First, are self‐similar dynamics exhibited by movements during affordance judgments of aperture pass‐through‐ability? Second, if self‐similar dynamics occur during the task, how do they compare across the three modalities (i.e., judgments made with vision, rod, or Enactive Torch)? Third, if self‐similar dynamics do occur to a similar degree across the three modalities, two of which involve nonbiological tools outside the body periphery, does that mean participants become person‐plus‐tool systems in order to perform the task? We hypothesized that participants’ arm movements while wielding haptic tools during the affordance‐judgment task would exhibit self‐similar dynamics. Participants’ eye movements were not recorded. However, we appeal to previous research demonstrating that eye movement structure—by nature of the visual system—exhibits self‐similar dynamics during visual‐search tasks (e.g., Aks, Zelinsky, & Sprott, 2002; Stephen & Anastas, 2011).

This work expands on previous research by the authors, including the finding by Favela and colleagues (Favela, Riley, Shockley, & Chemero, 2018) that participants’ perception of the boundary between aperture widths that were pass‐through‐able versus non‐pass‐through‐able were statistically equivalent across sensory modalities. In other words, participants made judgments regarding the affordance “pass‐through‐able” that were of equivalent accuracy whether they perceived with vision, haptically via a rod, or haptically via the Enactive Torch. Moreover, when grouped together, the two haptic tools were statistically more accurate than vision. Despite performing better with the haptic tools, participants were less confident in their judgments using the rod and Enactive Torch as compared to vision. The results motivate the need to assess SSD in the context of functional behaviors, for example, to support the development of prosthetics and tools for the visually impaired (Favela et al., 2018; Travieso, Gomez‐Jordana, Diaz, Lobo, & Jacobs, 2015). Findings from the perceptual judgments provide preliminary support for extended cognition, they do not alone provide direct support. In this paper, we bolster the case for extended cognition by expanding on the perceptual judgment findings by way of analyses focused on movement dynamics during tool use. We do so by highlighting a quantitative framework for assessing the degree of functional information exchange between humans and tools, where high‐functioning single systems are defined by self‐similar dynamics. Moreover, we aim to strengthen our case by assessing multiple forms of self‐similar dynamics: monofractal, multifractal, and recurrence.

## 2 Method

**Fig 1:**
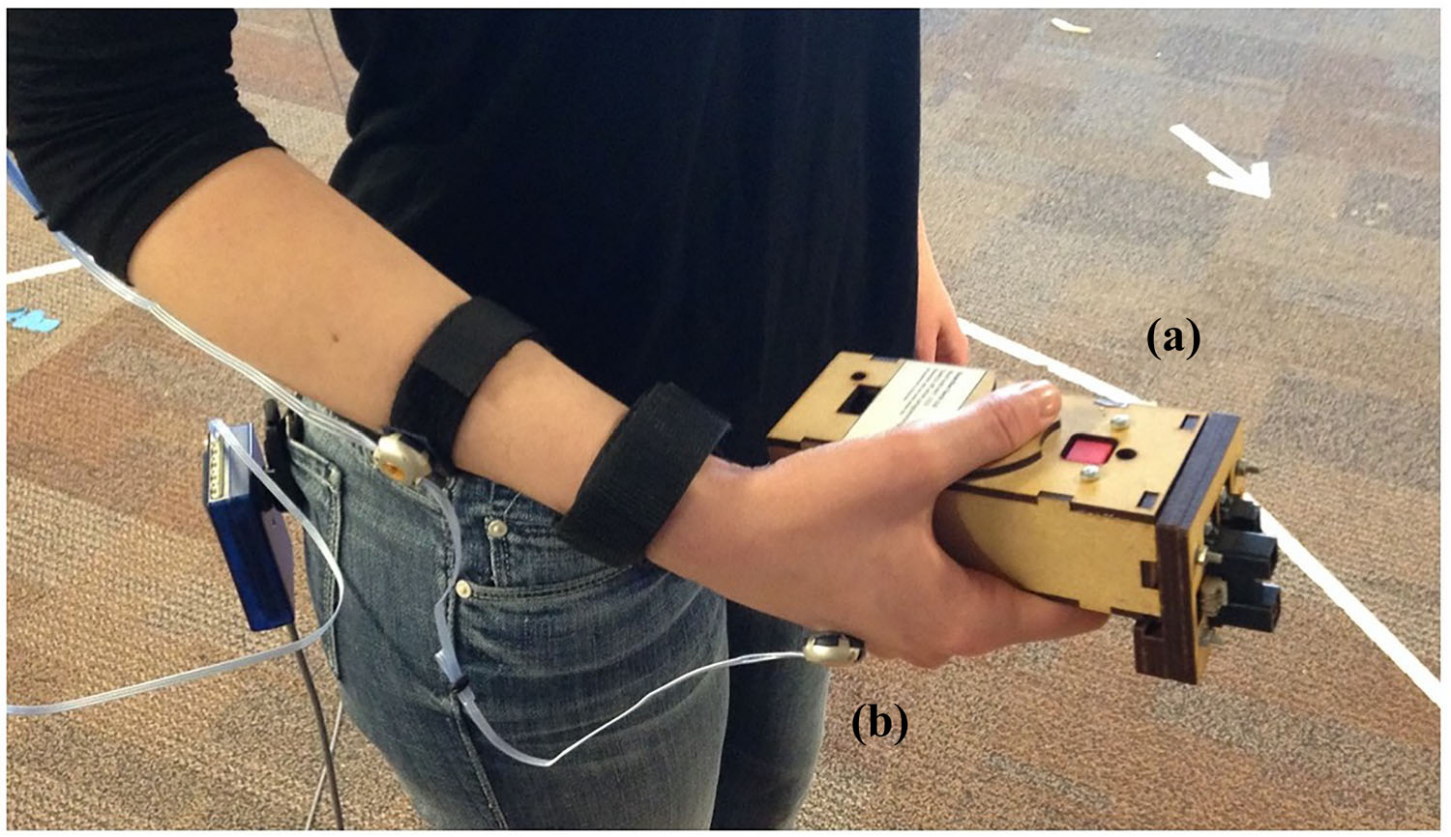
Enactive Torch and motion‐capture setup. *Note*. The Enactive Torch version 5 was utilized in the experiment (a). The infrared range sensors are at the front (pointing right in image). The device connects via a cord to a vibrational motor attached by a Velcro strap to the user's wrist. The Enactive Torch was designed by Tom Froese and Adam Spiers (2007). Body kinematics were tracked with a motion capturing system (Optotrak; Northern Digital, Inc.). Participants’ movements were tracked via a “marker” attached to the top of the hand that wielded the rod and Enactive Torch (b).

Here, we summarize the experiment that provided the currently analyzed movement data (for full details, see Favela et al., 2018). Undergraduate students (27; 19 women and eight men) from a large university in the Midwestern United States participated. They reported no experience with handheld mobility assistance devices (e.g., crutches) or a history of movement or perception disorders. Informed consent was provided by participants after being presented with an overview of the experimental procedure and the Institutional Review Board‐approved (# 11041501E) study procedures and consent document. During trials, participants were placed in front of an aperture that consisted of a doorway with two sliding panels that could slide laterally to create aperture widths ranging from 40 to 75 cm. Participants made affordance judgments of pass‐through‐ability utilizing SSD in two of the three conditions: a cane‐like wooden rod (length: 121.5 cm, diameter: 1.27 cm; weight: 113.4 g) and the Enactive Torch (length: 15.8 cm; width: 5.8 cm; height: 4.6 cm; weight: 350 g; Fig. 1). By moving the Enactive Torch (e.g., side to side, up and down, etc.), the user obtains information about the layout of nearby surfaces. Distance is detected with infrared range sensors and translated to haptic (vibrational) stimuli by a motor embedded in the wrist strap worn by the user. The intensity of vibration is inversely proportional to the distance of a surface, such that more intense vibrations are caused by near surfaces. Participants wore earmuffs (3M PELTOR Sport Bull's Eye 9) in all three conditions and an additional blindfold in the two haptic conditions.

All participants stood with the heels of their feet 76 cm from the aperture and were told they would remain at this same distance from the aperture for the entire experiment. Participants were presented each of the three modality conditions in blocks that were presented in random order across participants: (a) vision, (b) blindfolded with a rod, and (c) blindfolded with Enactive Torch. Each condition block consisted of 16 aperture widths: eight widths at 5‐cm increments between 40 and 75 cm randomly presented once and then randomly presented again. Participants were instructed to provide perceptual reports concerning their ability to pass through the aperture by responding “yes” or “no” with regard to whether they could comfortably walk—that is, without rotating the shoulders or hitting the panels with their arms—through the presented aperture on a given trial. Participants were asked to provide confidence ratings between 1 and 7 (1: “*not confident in my judgment*”; 7: “*very confident in my judgment*”) of their “yes” or “no” responses. In the vision condition, participants were asked to close their eyes between trials and to point the rod and Enactive Torch to the side between trials in the haptic conditions while panels were moved to create the aperture width for the next trial. Body kinematics were tracked with a motion‐capturing system (Optotrak; Northern Digital, Inc.) and data were collected via First Principles software. Participants had a “marker” placed on the top of the hand they used to wield the rod and Enactive Torch, with movements recorded at a frequency sample of 100 Hz.

## 3 Analyses

Next, we outline three complementary analytic approaches for understanding movement dynamics during person‐plus‐tool interactions: detrended fluctuation analysis (DFA), multifractal DFA (MFDFA), and auto‐recurrence quantification analysis (aRQA). First, we utilized DFA to examine the self‐similar dynamics of movement during tool use in terms of the Hurst exponent (*H*), a linear relation indicating the degree of self‐similarity across scaling. Doing so is central to the research question examining whether human‐plus‐tool interactions can be qualified as single systems.

Because it was plausible for both Enactive Torch and rod trials to exhibit self‐similar dynamics, we examined more detailed information regarding differences in movement between Enactive Torch and rod via MFDFA. In addition to providing the monofractal *H* value (similar to DFA), MFDFA supplies a range of *H* values that indicate the degree to which self‐similarity varies across the time series and based on timescale. MFDFA has been used to determine the relative health and functioning of a system, for example, age‐related decline in brain‐related dynamics, effectively identifying heart disease, and muscle fatigue (Ivanov et al., 1999; Makowiec et al., 2011; Suckling, Wink, Bernard, Barnes, & Bullmore, 2008; Wang, Ren, Li, & Wang, 2007). Self‐similar dynamics (e.g., identified via DFA) are widely implicated as marks of healthy systems. However, there is no hard‐and‐fast rule as to whether low versus high *variability* in self‐similarity is ideal (e.g., identified via MFDFA). That is, both increased (Song, Lee, Kim, Lee, & Kim, 2005; Wang et al., 2007) and decreased (Ivanov et al., 1999) variations in self‐similar structure have been associated with negative health and performance outcomes. One possibility is that the benefits associated with specific timescales of influence on signal structure are context‐specific. For example, the dominance of fast timescale fluctuations may be healthy in one system but either overwhelm or be inconsequential for another system where slow timescales are key to performance (Van Orden et al., 2012). Another possibility is that the benefits of greater or lesser variation in self‐similarity differ based on the degree to which the spectrum overlaps with *H* in the range of fractal scaling, where a narrowed spectra in the range of white noise (relatively random) has negative effects on the system. Although more research is needed to examine differences in functioning associated with high and low variability in self‐similar structure, MFDFA has been fruitfully applied in a variety of contexts and provides a more nuanced perspective of self‐similarity than DFA alone.

Last, we utilized aRQA to describe the degree to which novel versus repetitive movements were generated during each trial with Enactive Torch and rod. Nonlinear dynamical systems theory (and, relatedly, complex systems theory) highlights that well‐functioning systems must be both adaptable and stable—not too rigid or too random—such that these opposing forces are balanced. Systems that exhibit a functional balance between order and disorder are called “far from equilibrium,” which is considered a universal feature of healthy systems (Bossomaier & Green, 2007). The aRQA is a means for capturing this balance between repetition (i.e., stability) versus novelty (i.e., adaptability) within a signal, where prior research highlights the tradeoff in higher repetition corresponding to greater degrees of functional coordination (e.g., Richardson & Dale, 2005) and lower repetition indicating the adaptive ability to re‐organize behavior to meet task demands (e.g., Amon, Vrzakova, & D’Mello, 2019). Thus, we hypothesize that participants will exhibit a balance between repetitive and novel movement patterns with both tools. Furthermore, it is expected that more coordinated, repetitive movements will be produced with the rod, which is a type of tool people are more familiar with, as opposed to the more novel Enactive Torch.

### 3.1 DFA

**Fig 2:**
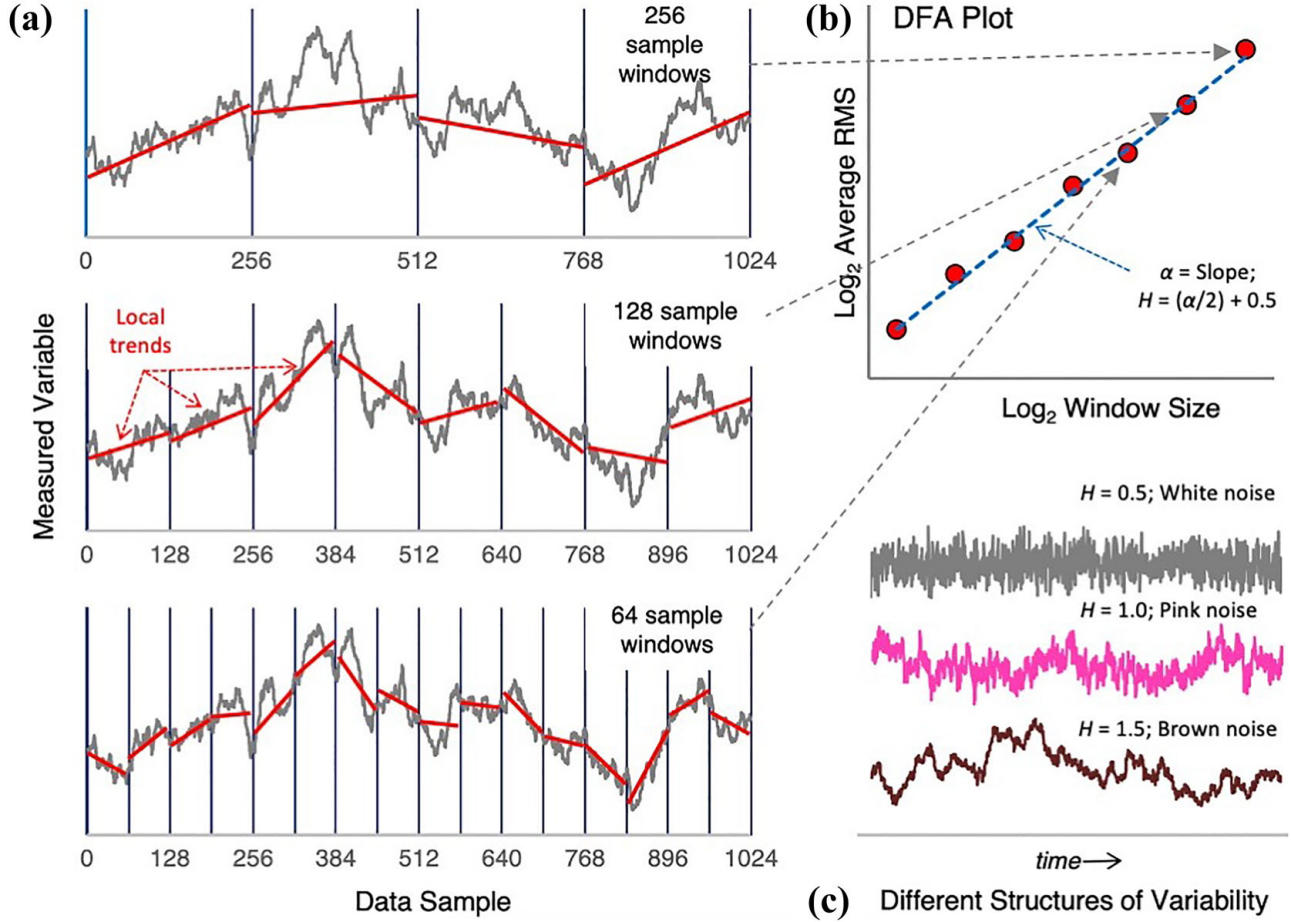
Detrended fluctuation analysis (DFA). *Note*: Example DFA procedure. (a) Linear detrending occurring at powers of two: 64 samples (bottom), 128 samples (middle), 256 samples (top). The log–log plot of the remaining trend for each window size along a slope or alpha (*α*). (b) The *α* value is transformed to obtain the Hurst (*H*) value in order to reveal degrees of self‐similarity in a signal. Example *H* values: *H* ≈ 0.5 indicates white noise (random or unstructured signal), *H* ≈ 1 indicates pink noise (fractal or highly structured), and *H* ≈ 1.5 indicates brown noise (Brownian motion or local randomness with long‐term structure). (c) (Modified and reprinted with permission from Rigoli et al. (2020). CC BY 4.0.).

DFA was employed to assess for self‐similarity in the body kinematic data recorded by the motion‐capturing system during trials with the two haptic SSDs. DFA detects statistical self‐similarity after removing local trends within specified windows of time in a dataset (Peng et al., 1994). After this linear detrending, the remaining data represent fluctuations around a global trend. For each window size, the log–log plot of the transformed frequency as a function of the transformed amplitude fluctuations reveals a linear relation indicating the degree of self‐similarity across scaling, given by the Hurst exponent (*H*; Fig. 2). Accordingly, DFA is an appropriate method for assessing fractal structure (Delignieres et al., 2006; Holden, 2005).

For the present study, three types of dynamics are pertinent. *H* exponents near one (*H* ≈ 1) indicate pink noise, also referred to as 1/*f* scaling or 1/*f* noise. Pink noise reveals a signal's fractal structure or self‐similar temporal or spatial patterns across scales (Holden et al., 2009; Kello, Beltz, Holden, & Van Orden, 2007). Pink noise contrasts with white noise, which represents random or independent timesteps (*H* ≈ 0.5). *H* exponents close to 1.5 or higher (*H* ≈ 1.5) represent brown noise, or Brownian motion, which describes patterns of variability that exhibit a random walk pattern that has the appearance of local randomness or independence but global structure (Gilden, 2001; Holden, 2005). Brownian noise often describes movements of natural systems where it is not easy to predict a specific movement trajectory, but the trajectory is always dependent on the system's previous position.

Consistent with Ihlen (2012), our DFA procedure analyzed data in increments of integer powers of two, such as 512, 1024, 2048, and so forth. In line with this criterion, a minimum cutoff of 1024 data points was set. For trials where data had gaps shorter than 20 ms, a MATLAB (MathWorks) function was used to interpolate those missing sections of the time series using a polynomial fit. Trials were excluded if no data were collected for reasons such as the marker being outside of the motion capturing range. After applying these criteria, data from 19 of the original 27 participants remained viable for analysis.

DFA parameters were set in accordance with literature standards (e.g., Cannon, Percival, Caccia, Raymond, & Bassingthwaighte, 1997; Eke, Herman, Kocsis, & Kozak, 2002; Ihlen, 2012), which the authors have utilized in prior research (e.g., Amon et al., 2018; Dotov et al., 2010; Favela, Coey, Griff, & Richardson, 2016). The analysis was set to evaluate the first 1024 data points per time series after each trial began. Data integration was not needed as the data were already fractional Brownian motion and appropriate for DFA. We utilized a linear detrend, 50% window overlap, minimum window size of 4, and maximum window size of 128 (i.e., 1024 data points/8 = 128). Researchers can opt to remove linear, polynomial, cubic, and other trends from the data, where linear detrending is standard for time series with a smaller minimum window size to avoid overfitting of the polynomial trend (Ihlen, 2012). In addition to these parameters, MFDFA included the following parameters: *q* minimum = –5; *q* maximum = 5; and *q* step = 2.

### 3.2 MFDFA

As stated above, a fractal displays the same structure at various spatial or temporal scales or scale invariance. Traditional (mono)fractal analyses such as DFA are defined by a single power‐law exponent and assumes that such scale invariance is independent in time and space (Ihlen, 2012). However, there can be spatial and temporal variations within a signal structure. Just as means and standard deviations may vary within a time series and for events of different magnitudes, it follows that fractal structure can also vary across the time series. *Multifractal* analysis characterizes different scaling properties across a signal, capturing nonlinear cascades with sporadic bursts of activity. In doing so, multifractal analysis indicates the relative influence of different timescales in determining time series structure (Ihlen, 2012).

MFDFA accounts for nonlinear cascades by adding a *q* parameter to the DFA, which weighs the influence of small and large fluctuations (i.e., root‐mean‐square; RMS). RMS of variation around local trends is raised to the value of each *q* parameter. The more negative the *q‐*value, the more strongly influenced the segment is by small fluctuations; the more positive the *q‐*value, the more influenced the segments are by large fluctuations; *q*s of 0 are neutral to the influence of relatively small or large fluctuations. The variation of *H* exponents based on *q* provides an index of *multifractality* or the degree to which 1/*f* scaling varies across the signal (Ihlen, 2012). Put simply, MFDFA generates numerous *H* values, with each *H* value emphasizing different scales of interest. More extreme fluctuations or “bursts” of activity in the time series are weighted using the *q*‐value, which, in turn, influence the *H* exponent.

We utilized *q*‐values ranging from –5 to 5 in increments of 2, in order to examine the relative amplitude of different timescale fluctuations within the movement time series. In doing so, we examine the singularity spectrum of the signal, or the range of *H* values that define the signal across timescales, where a larger width corresponds to a greater deviation in 1/*f* scaling based on small and large fluctuations. We also examine the relative *H* minima and maxima of the time series in order to examine the degree to which fast and slow timescales influence the structure of the signal, respectively.

Notably, MFDFA produces the same *H* value derived from DFA, though researchers who report MFDFA tend to focus on additional information derived from the analysis (esp. *H* spectral width). As previously noted, the *q* parameter weights different scales of the time series before calculating each subsequent *H* value. When the *q*‐parameter is 0, it is neutral to the influence of small or large RMS variability and derives a monofractal *H* value identical to DFA. Thus, just as the *H* minimum (e.g., at *q* = –5), *H* maxima (e.g., at *q* = 5) and their difference (*H* spectral width) are of interest when conducting MFDFA, the monofractal *H* value at *q* = 0 remains of interest. It follows that monofractal and MFDFA analyses are not mutually exclusive, and we discuss *H* values at various *q*‐value in the spectra to better understand movement dynamics during the experiment.

For the current work, both DFA and MFDFA employed MATLAB (MathWorks) functions utilized in prior research of cognitive systems (original code versions developed by Charles Coey based on Ihlen, 2012). The data source was the *x*‐axis (i.e., side‐to‐side or left‐to‐right) motion of participants during each trial while they used either the rod or Enactive Torch to inform judgments of aperture pass‐through‐ability.

Utilizing DFA in human behavior research is becoming more common (see references in Section 3.1). Though there has been some application (e.g., Davis, Brooks, & Dixon, 2016; West & Scafetta, 2005), the same is not true of MFDFA. Here, we expand on the literature utilizing MFDFA in human behavior research by examining the relative scales of fluctuation during person‐plus‐tool movement dynamics.

### 3.3 aRQA

Recurrence quantification analysis captures the temporal evolution of behavior by identifying the degree to which a signal returns to previous states, providing an index of repeat or “recurrent” values over time (Marwan, Romano, Thiel, & Kurths, 2007; Webber & Marwan, 2015). Although there are different variations of RQA that capture alignment between two signals (cross‐RQA; Coco & Dale, 2014) or numerous signals (multidimensional‐RQA; Wallot, Roepstorff, & Mønster, 2016), auto‐RQA (aRQA; Webber & Zbilut, 2005) measures repetitiveness within one time series and is appropriate for characterizing movement trajectories of an individual wielding a rod or Enactive Torch. Though they are not typically utilized in conjunction, analyses such as DFA and RQA are well‐suited for application to many of the same phenomena, especially if self‐similarity is of interest. The reason is that, by definition, fractals are recurrent: They are self‐similar structures that repeat at different scales (Webber, 2012). aRQA supplements DFA and MFDFA by identifying the relative balance between repetition versus novelty, where such a balance is foundational to healthy systems (see Section 3).

aRQA first produces a distance matrix that indicates the Euclidean distance between all time points within a time series, where larger values indicate greater differences in values between two time points. A radius is applied to the distance matrix, with distance values less than the radius constituting a recurrent point (1) and distance values greater than the radius constituting a non‐recurrent point (0). This binarizes the recurrence matrix—a qualitative depiction of recurrence—such that recurrent points are highlighted in black. Although a number of measures can be derived from the RQA matrices, here we focus on recurrence rate, or the percentage of recurrent points within the matrix, excluding the diagonal line of identity where the time series is recurrent with itself at lag 0.

RQA requires that a number of parameters be selected prior to analysis. First, phase‐space reconstruction is sometimes recommended in order to uncover the underlying dimensions of a given signal (see Webber & Zbilut, 2005). However, uncovering dimensionalities of the movement signals was not of primary interest to the present research, motivating us to avoid an unnecessary transformation of the measured signal. In addition, we are not aware of research that has tested the assumption that phase‐space reconstruction methods are robust in recovering true underlying dimensionalities of individual signals (e.g., creating delayed copies of a heart rate variability signal accurately captures its subprocesses). Without phase space reconstruction, RQA maintains its purpose of identifying points of recurrence within the original time series, indicating whether two points in time are sufficiently similar to one another to be counted as recurrent. For these reasons, we set the embedding dimension and delay parameters to one. The radius is traditionally set to maintain an average recurrence rate across signals that are relatively low, for example, between 2% and 5% (e.g., Coco & Dale, 2014). This is necessary to avoid mischaracterizing very different values as being similar to one another, as well as to avoid ceiling effects where a large radius might result in some participants having almost all recurrent points. For the rod, we maintained an average recurrence rate of 4.92 (*SD* = 1.39) and 4.70 for the Enactive Torch (*SD* = 1.75) using a radius of .04. MATLAB (MathWorks) code from Wallot et al. (2016) was utilized to analyze movement dynamics from each trial. Mixed‐effects models were completed in R (R Core Team, 2018) using the lme4 package (linear mixed‐effects models using “Eigen” and S4; https://cran.r‐project.org/web/packages/lme4/index.html).

## 4 Results

### 4.1 1/f scaling

Summary statistics for DFA and MFDFA based on condition are presented in Table 1. Each participant was assigned an average score for each variable before summary statistics were calculated across participants. Given that participants varied in the number of trials utilized in the current analyses, examining averaged participant scores was necessary to avoid weighting participants with a greater number of trials more than others.

**Table 1:** Summary statistics of detrended fluctuation analysis (DFA) and multifractal DFA analyses

	*H*	*H* Spectral Width	*H* Minima	*H* Maxima
Condition	*M* (*SD*)	*M* (*SD*)	*M* (*SD*)	*M* (*SD*)
Enactive Torch	1.27 (.09)	1.52 (.14)	0.87 (.11)	2.4 (.09)
Rod	1.29 (.08)	1.35 (.15)	0.94 (.09)	2.29 (.19)

DFA yielded *H* values indicative of 1/*f* scaling for movements of both the rod (*M* = 1.29, *SD* = 0.079) and Enactive Torch (*M* = 1.27, *SD* = 0.09) during perceptual judgments. The findings indicate that both rod and Enactive Torch wielding produced movement patterns squarely between 1/*f* scaling (*H* = 1) and Brownian motion (*H* = 1.5), consistent with the notion of finding self‐similar fractal structure within movement data. Specifically, values in this range (Holden, 2005, p. 298) are consistent with a particular type of movement data or global trend referred to as anti‐persistent Brownian motion, where successive increments in a time series tend to have opposite signs (e.g., positive followed by negative change). The fact that a self‐similar structure is embedded within an anti‐persistent trend is intuitive, in which anti‐persistence reflects the back‐and‐forth motion participants tended to use when exploring the aperture width with the rod or Enactive Torch.

### 4.2 Variation in 1/f scaling

A mixed‐effects multiple linear regression model was utilized to examine the extent to which DFA *H* values for each trial varied as a function of condition (rod or Enactive Torch), trial number, pass‐through‐ability judgment, self‐reported confidence in judgment, and aperture width, with the participant as a random intercept. The number of trials available for each participant was included as a covariate to control for differences between participants. The results are shown in Table 2, Model 1. Findings indicate that while *H* values differed significantly across participants based on the random intercept, all other predictors were non‐significant, *p* > .10. Put differently, *H* values were statistically equivalent regardless of the instrument used or the aperture width. *H* was also relatively stable regardless of pass‐through‐ability and confidence judgments. Notably, order effects were not present, indicating that movement dynamics were similarly fractal throughout the trials.

**Table 2:** Monofractal *H* value (Model 1), multifractal spectrum (Model 2), *H* minima (Model 3), and *H* maxima (Model 4) based on condition, task features, and participant judgments

	Dependent Variable:					
	Hurst (*H*)			Spectral Width		
	*β*	*CI* (95%)	*p*	*β*	*CI* (95%)	*p*
Condition (ET)	–.03	(–0.08, 0.01)	.13	.08	(0.005, 0.16)	.04
Trial number	–.001	(–0.003, 0.002)	.58	–.002	(–0.01, 0.002)	.26
Aperture width	–.0002	(–0.003, 0.002)	.90	.01	(0.004, 0.01)	<.001
Judgment (yes)	–.01	(–0.08, 0.05)	.66	–.08	(–0.18, 0.03)	.14
Judgment confidence	–.01	(–0.02, 0.01)	.37	–.01	(–0.03, 0.02)	.65
Trials available	.01	(–0.01, 0.03)	.28	–.01	(–0.03, 0.01)	.40
Random intercept: Participant	1.18	(0.79, 1.56)	<.001	1.22	(0.73, 1.71)	<.001
Observations		344			344	
Akaike inf. crit.		–8.81			332.43	
Bayesian inf. crit.		25.75			367.00	

	Dependent Variable:					
	*H* Minima			*H* Maxima		
	*β*	*CI* (95%)	*p*	*β*	*CI* (95%)	*p*
Condition (ET)	–.05	(–0.11, 0.004)	.08	.03	(–0.03, 0.09)	.33
Trial number	.001	(–0.002, 0.005)	.36	–.001	(–0.004, 0.002)	.59
Aperture width	–.0001	(–0.003, 0.003)	.97	.01	(0.004, 0.01)	<.001
Judgment (yes)	–.01	(–0.09, 0.07)	.85	–.07	(–0.16, 0.01)	.09
Judgment confidence	–.01	(–0.03, 0.01)	.37	–.02	(–0.04, 0.004)	.12
Trials available	.01	(–0.01, 0.03)	.48	–.003	(–0.01, 0.01)	.53
Random intercept: participant	.81	(0.31, 1.30)	.002	2.11	(1.80, 2.42)	<.001
Observations		344			344	
Akaike inf. crit.		159.67			168.42	
Bayesian inf. crit.		194.24			202.99	

*Note*. Standardized estimates (*β*) and 95% confidence intervals (CI) for a mixed‐effects multiple linear regression model examining monofractal *H* value, multifractal spectrum, *H* minima, and *H* maxima as a function of aperture task features and participant judgments.

We used a series of mixed‐effects multiple linear regression models to examine the extent to which multifractal dynamics—including spectral width, *H* minimum, and *H* maximum—varied based on condition (rod or Enactive Torch), trial number, pass‐through‐ability judgment, self‐reported confidence in judgment, and aperture width, with the participant as a random intercept and controlling for the number of trials available for each participant.

First, we found that MFDFA spectral width varied significantly based on condition (rod or Enactive Torch), with Enactive Torch exhibiting a wider *H* spectrum (*β* = .08, *p* = .04). In addition, greater aperture widths corresponded to wider *H* widths (*β* = .01, *p* < .001). MFDFA *H* width did not significantly vary based on the trial number, degree of self‐reported judgment confidence, or perceived pass‐through‐ability, *p* > .10. See Table 2, Model 2, for a summary of results.

**Fig 3:**
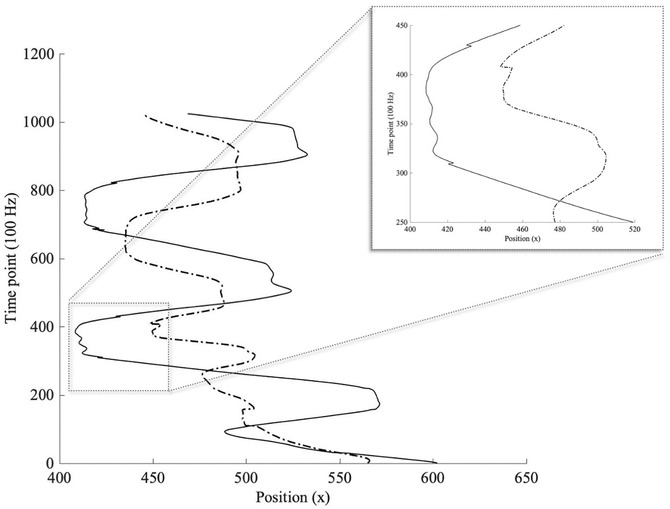
Time series movement examples of Enactive Torch and rod. *Note*. Example Enactive Torch (solid line) and rod (dash‐dotted line) time‐series movements. Back‐and‐forth movements are exhibited while wielding both tools when participants could presumably sense the edges of the aperture opening. The zoomed‐in portion provides a qualitative depiction of lower *H* minima associated with the Enactive Torch, which suggests that participants demonstrated somewhat higher amplitude movements at faster timescales as compared with the rod.

Second, we examined the extent to which *H* minimum varied as a function of the same set of predictors as in the previous model (Table 2, Model 3). Findings indicated that condition (rod or Enactive Torch) was a significant predictor of the *H* minimum. Specifically, the Enactive Torch was marginally associated with lower *H* minima, suggesting that participants demonstrated somewhat higher amplitude movements at faster timescales, as compared with the rod (*β* = –.05, *p* = .07).^4^ Figure 3 illustrates this concept qualitatively through comparison of example Enactive Torch (solid line) and rod (dash‐dotted line) time series, where short back‐and‐forth movements were made when participants could presumably sense the ends of the aperture by quickly waving the Enactive Torch back‐and‐forth. Findings suggest that the Enactive Torch condition was associated with more fine motor movements than the rod.^5^ *H* minima were not significantly associated with the trial number, aperture width, perceived pass‐through‐ability, judgment confidence, or the number of trials available for analysis, *p* > .10.

**Fig 4:**
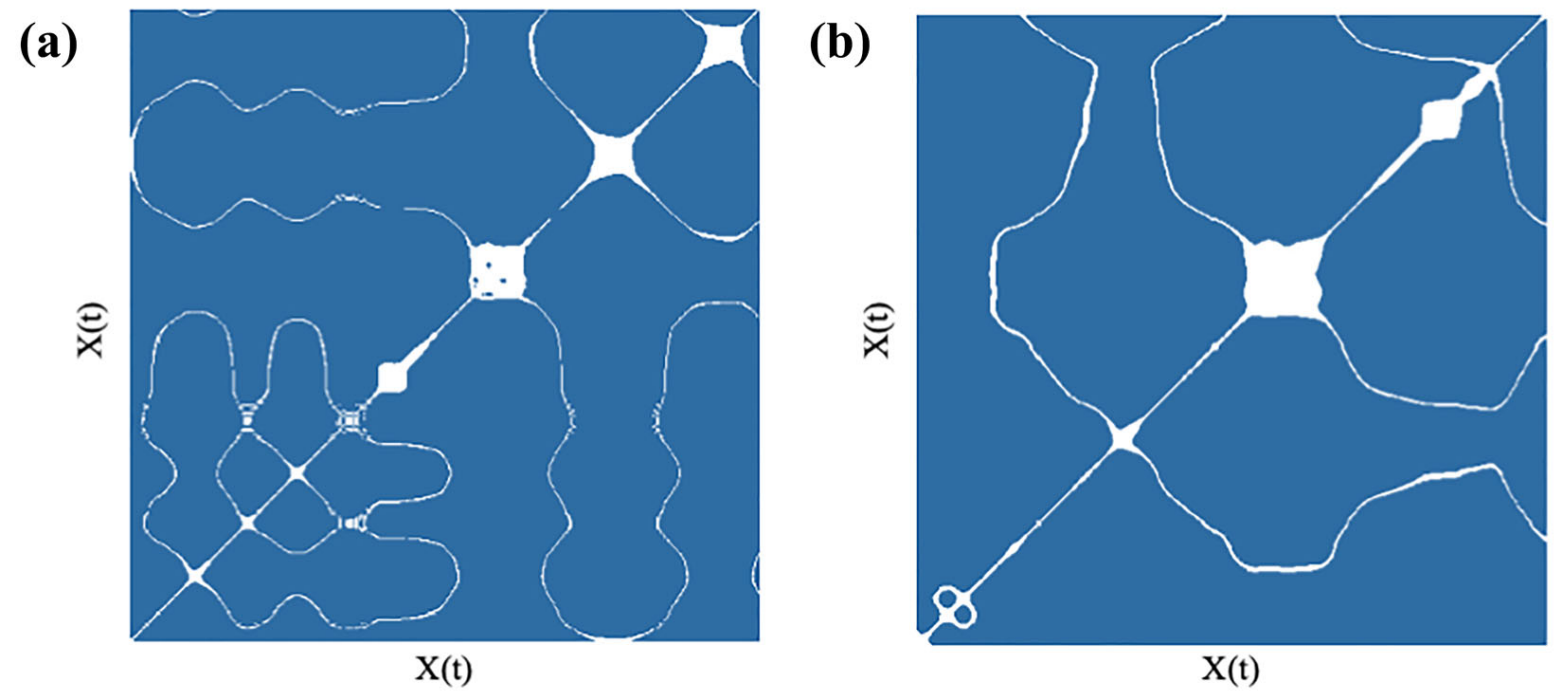
Recurrence plot examples of arm movements while wielding Enactive Torch and rod. *Note*. Example Enactive Torch (a) and rod (b) recurrence plots depicting the auto‐recurrence quantification analysis results demonstrating that participants wielding the Enactive Torch exhibited less recurrence and explored more space (a), while participants wielding the rod exhibited more recurrence but explored less space (b).

Third, we examined the extent that the same set of predictors as in the previous two models predicted MFDFA *H* maxima. The results are shown in Table 2, Model 4. We found that higher *H* maxima were associated with larger aperture widths, indicating higher amplitude movements at slower timescales (*β* = .008, *p* < .001). In addition, positive pass‐through‐ability judgments—when participants responded that an aperture was pass‐through‐able—were marginally associated with lower *H* maxima (*β* = –.07, *p* = .09). MFDFA *H* maxima did not significantly vary as a function of condition, trial number, or self‐reported judgment confidence, *p* > .10.

### 4.3 aRQA

A mixed‐effects multiple linear regression model was utilized to examine the extent to which recurrence rate of movement dynamics during each trial varied based on condition (rod or Enactive Torch), trial number, pass‐through‐ability judgment, self‐reported confidence in judgment, and aperture width, with the participant as a random intercept. The number of trials available for each participant was included as a covariate to control for differences between participants. The findings indicated that participants wielding the Enactive Torch exhibited less recurrence and therefore explored more of the space as compared to when participants wielded the rod (*β* = –.37, *p* = .02). Unlike with the DFA and MFDFA models, the trial number was significantly associated with recurrence rate, such that there was greater recurrence during later trials (*β* = .03, *p* < .001). Marginal effects are also present, suggesting narrower aperture widths may correspond to greater recurrence (*β* = –.02, *p* = .06). In addition, a marginally significant effect suggests that positive judgments of pass‐through‐ability predicted higher recurrence rates (*β* = .38, *p* = .09). Recurrence rate was not significantly related to participants’ judgment confidence or the number of trials available for analysis, *p* > .10. See Table 3 for detailed results.

**Table 3:** Movement recurrence based on condition, task features, and participant judgments

	Dependent Variable:		
	Recurrence Rate		
	*β*	*CI* (95%)	*p*
Condition (ET)	–.37	(–0.69, –0.05)	.03
Trial number	.03	(0.02, 0.05)	< .001
Aperture width	–.02	(–0.04, 0.001)	.07
Judgment (Yes)	.38	(–0.05, 0.82)	.09
Judgment confidence	.03	(–0.08, 0.13)	.64
Trials available	.03	(–0.02, 0.07)	.22
Random intercept: participant	4.48	(3.02, 5.94)	<.001
Observations		344	
Log‐likelihood		–634.43	
Akaike inf. crit.		1286.86	
Bayesian inf. crit.		1321.43	

*Note*. Standardized estimates (*β*) and confidence intervals (CI) for a mixed‐effects multiple linear regression model examining the relationship between recurrence rate and aperture task features and participant judgments.

### 4.4 Participant characteristics

Although not central to the present research questions, the previously presented models revealed that fractal dynamics significantly varied across individual participants as indicated by the random intercept. Thus, we conducted an a posteriori mixed‐effects multiple linear regression analysis to examine the extent to which individual characteristics of the participants predicted 1/*f* scaling. Specifically, we regressed *H* value on participants’ aperture‐to‐shoulder width, age, gender, race, and weight. Although there was a marginally significant effect indicating that higher weight corresponded to lower *H* values (*β* = −.01, *p* = .08), participant characteristics identified during the experiment were overall non‐significant in predicting differences in 1/*f* scaling, *p* > .10. See Table 4 for full results.

**Table 4:** *H* value based on participant characteristics

	Dependent Variable:		
	Recurrence Rate		
	*β*	*CI* (95%)	*p*
Aperture‐to‐shoulder ratio	.57	(–1.00, 2.14)	.48
Age	.01	(–0.07, 0.09)	.82
Gender (male)	.20	(–0.22, 0.62)	.36
Weight (lbs)	–.01	(–0.01, 0.001)	.08
Random intercept: participant	1.13	(–1.59, 3.84)	.42
Observations		345	
Log‐likelihood		31.27	
Akaike inf. crit.		–48.54	
Bayesian inf. crit.		–21.63	

*Note*. Standardized estimates (*β*) and confidence intervals (CI) for a mixed‐effects multiple linear regression model examining the relationship between monofractal *H* value and participant characteristics.

## 5 Discussion

Utilizing methods from dynamical systems theory, we quantified participants’ dynamics to test our hypothesis that arm movements would exhibit self‐similar dynamics while wielding two haptic SSDs during an affordance‐judgment task. Consistent with this hypothesis, DFA yielded values indicative of self‐similarity or 1/*f* scaling. A mixed‐effects multiple linear regression model reinforced the DFA results by indicating that 1/*f* scaling was stable across participants regardless of trial order, aperture width, as well as affordance and confidence judgments. Notably, the absence of a trial order effect (i.e., learning effects) suggests that participants readily incorporate rod and Enactive Torch tools into the system used during the perceptual judgment task. As discussed in previous research by the authors (Favela et al., 2018), the absence of a trial order effect (i.e., learning effects) suggests that perceptual systems (i.e., human participants) have the ability to flexibility achieve goals with their body in various ways, which includes incorporating objects as functional components. If perceptual systems are of such a highly adaptable kind, then it would suggest that human perceptual systems are extended cognitive systems of the soft‐assembled kind. Soft‐assembled systems are not hardwired or preprogrammed for limited ranges of particular outputs (Favela, 2019; Favela et al., 2018; Thelen & Smith, 2006). Extended cognitive systems qua soft‐assembled systems comprised parts that can temporarily coordinate in various ways to achieve tasks.

Although 1/*f* scaling was generally stable across conditions, trials, task, and participant features, multifractal analyses indicated that Enactive Torch movements exhibited a greater spectral width than the rod movements. The findings suggest that the Enactive Torch facilitated a range of exploratory movements not observed with the rod. In particular, this effect appears to be driven primarily by higher amplitude, or faster timescale, fluctuations in movement. Thus, the Enactive Torch may promote a greater range of motion, especially in terms of fine motor movements. Corroborating evidence is provided by the aRQA results that indicate the Enactive Torch was associated with less repetitive motions or, put differently, more novel positioning of the arm during tool use. The strength of the findings is further supported by the presence of additional intuitive effects, for example, that higher amplitude movements at slower scales occurred when participants made judgments of apertures with wider widths. That is to say, wider aperture widths were associated with larger sweeping movements.

aRQA findings also indicate that movement dynamics with both SSDs exhibited a balance between repetitive and novel movement patterns, with more coordinated, repetitive movements associated with the rod. In addition to more novel fine motor movements evidenced with MFDFA, this may have occurred as the rod is a type of tool people are more familiar with, such that rod use produced more predictable patterns as opposed to more exploratory patterns being produced with the more novel Enactive Torch. Additional research is needed to determine whether the more novel patterns produced during Enactive Torch use would lessen with experience. Overall, the precise reasons why the Enactive Torch provides a greater range of motion than a rod is worth further investigation. It can be argued that because the Enactive Torch is shorter in length than the rod (15.8 and 121.5 cm, respectively), it is easier to wield. Yet the Enactive Torch weighs more than double of what the rod does (350 and 113.4 g, respectively), which would seem to—intuitively—result in a more limited range of motion. The interplay of an object's various properties, such as size and weight, and their effect on their ability to be wielded intersects with issues of active and dynamic touch (e.g., Gibson, 1962; Michaels, Weier, & Harrison, 2007; Palatinus, Carello, & Turvey, 2011).

In the Introduction, we noted that with very few exceptions (e.g., Dotov et al., 2010) has extended cognition been directly empirically investigated. The current analyses contribute to the empirical case supporting the existence of extended cognition and provide an affirmative to the question, “Are person‐plus‐tool‐systems extended cognitive systems?” To be more precise, we have provided empirical support for the claim that person‐plus‐tool‐systems can exhibit *extended cognitive dynamics*; and in that way, they are cases of extended cognition. The affordance‐judgment findings suggest that participants made perceptually equivalent judgments across all three modalities: vision, rod, and Enactive Torch (Favela et al., 2018). Thus, based on verbal reports, the two SSDs are functionally equivalent to vision when it comes to the ability of participants to make judgments about the aperture pass‐through‐able affordance. Based on analyses of arm movement dynamics presented above, both the rod and Enactive Torch exhibited equivalent self‐similar dynamics during the exploratory behaviors that informed the judgments conveyed by verbal report. Though we did not record eye movements during this study, the findings are consistent with research demonstrating self‐similar movements during visual‐search tasks (e.g., Aks et al., 2002; Stephen & Anastas, 2011). Thus, we find functionally equivalent judgments and movement dynamics when using vision and SSDs (in the absence of vision) during perceptual judgment tasks.^6^

### 5.1 Limitations and future directions

The current work has a number of strengths with regard to previous research that has engaged with the topic of extended cognition. First, our hypothesis was directly about extended cognition. This differs from other literature that aimed to support extended cognition by appeal to research that could be interpreted as being about extended cognition but was not originally about that topic (for review see Wagman & Chemero, 2014). Second, this work can be supported by a variety of strong theoretical foundations, including, but not limited to, ecological psychology (Gibson, 1979/1986), enactivism (Thompson, 2007), and radical embodied cognitive science (Chemero, 2009). Third, and most importantly for our current aims, we provide a way to empirically assess extended cognition, which is an advancement on the typical tools for assessing it that have been limited to intuitions and thought experiments (cf. Adams & Aizawa, 2008; Clark & Chalmers, 1998). These strengths pave the way for additional experimental work on other forms of extended cognition (e.g., memory; e.g., Sutton, Harris, Keil, & Barnier, 2010) and broader forms of cognition as well (e.g., team cognition; e.g., Fiore & Wiltshire, 2016). That said, we are aware of a number of limitations of the current work. We discuss the limitations in relation to future research directions they motivate.

The first limitation—and perhaps a paradoxical one—of the current work is its novelty. That there are very few direct empirical assessments of extended cognition (e.g., Dotov et al., 2010), so too is it the case that none—as far as we know—have been replicated. Consequently, the empirical literature on extended cognition will remain suspect until replications of findings become more commonplace. Second, though we have claimed that the findings of our analyses of arm movement data are consistent with other studies of eye movement, the conditions of those studies were not the same as ours, namely, they did not involve affordance judgments (Aks et al., 2002; Stephen & Anastas, 2011). In view of that, findings from future research on extended cognition will be strengthened when multiple perceptual modalities and various cognitive extensions are assessed within the same experimental paradigm. Third, previous experimental work (e.g., Dotov et al., 2010) highlights the usefulness of perturbations in this type of research, for example, manipulating features of SSD, such as altering feedback from the Enactive Torch or adding weight to the rod. Fourth, although we did not find trial order effects on task performance, there are a number of worthwhile issues to explore concerning learning effects and other performance differences among various SSDs. There are clear differences between a rod (or cane) and the Enactive Torch, such as their physical dimensions, how they convey information about the environment via various effectors, and how perceptual calibration is structured by those effectors. Thus, additional research aimed at elucidating tool differences could elucidate their contributions to person‐plus‐tool systems.^7^ The fifth—and perhaps most substantial—limitation concerns the ability to arbitrate which theoretical approach best accounts for the data.

Theories and explanations in line with the received view in cognitive science state that perceptual judgments of the kind in the current work are explained by computations and representations that occur in the brain (e.g., Edelman, 1999; Stone, 2012). A common example of this approach is to understand tool‐use as involving representations of the tool becoming included in the brain's body schema, for example, as the left hand is represented in the primary somatosensory cortex, so too can a tool become represented (e.g., Jovanov, Clifton, Mazalek, Nitsche, & Welsh, 2015; Maravita & Iriki, 2004; Massen, 2013; Miller et al., 2019). Such computational‐representational‐brain‐based approaches to explaining tool‐use can include dynamics as key features of the explanations as well (e.g., Kuniyoshi & Sangawa, 2006; Kuniyoshi & Suzuki, 2004). On the one hand, such “neural‐body schema” approaches could be consistent with claims about extended cognition. For example, although a tool is represented in the somatosensory cortex, it could be the case that the computation includes features that extend beyond the brain, such as using an abacus to do arithmetic (Fischer & Brugger, 2011). On the other hand, such “neural‐body schema” approaches are commonly viewed as antithetical to extended cognition. This is the case because extended cognition is usually grouped with other theories that typically adhere to anticomputational and antirepresentational conceptions of cognition and that emphasize the roles of the body and environment, namely, “4E cognition”; that is, cognition as embodied, embedded, extended, and/or enactive (Newen, de Bruin, & Gallagher, 2018). The current work does not purport to settle any debates concerning the nature of cognition, action, or perception. We leave it to the reader to decide if the current findings support a computational‐representational‐brain‐based approach and are thus empirical evidence for the received view, or if it supports noncomputational, nonrepresentational, and/or nonbrain‐based views more akin to 4E cognition.

## 6 Conclusion

The current work lends support to extended cognition by demonstrating that the dynamics involved in affordance judgments can extend through the body and tools as perceptual judgments are made regarding action capabilities in an environment. Self‐similar dynamics indicative of a healthy and adaptive single system are robustly present when using SSDs such as the rod and Enactive Torch, across both tools and regardless of aperture width, trial order, participant characteristics, or participant judgments. Moreover, the dynamics of tool use can elucidate nuances of tool use, for example, its association with different types of exploratory movements that may prove to be functional in different task environments. In summary, we provide an empirical framework whereby self‐similar dynamics can be appealed to as tests of extensions of cognitive dynamics. In doing so, we demonstrate that person‐plus‐tool‐systems—in this case, SSD during affordance judgment tasks—can be appropriately characterized as extended cognitive systems.

## Conflict of interest

The authors declare no known conflict of interest to disclose.

## Notes

1 In contemporary analytic philosophy, there has been increased discussion as to whether there is a substantive distinction between questions of fact and questions of language (e.g., Chalmers, 2011; cf. Tollefsen & Dale, 2018, p. 276). Put more crudely, does the answer to a question depend on facts about the world or merely on the ways certain words are being used? We mention “verbal disputes” here to emphasize that our current aim is not to argue if the phenomenon we are investigating *is really* extended cognition or if it is *actually* embodied cognition, distributed cognition, and so forth. We are committed to our focus being extended cognition; though it could overlap with embodiment, and so forth.

2 We thank a reviewer for drawing our attention to other examples from the literature that they suggest are empirical studies of extended cognition (e.g., Auvray, Lenay, & Stewart, 2009; Richardson, Marsh, & Baron, 2007). It is worth reiterating that we do not claim that no empirical studies have ever been conducted on extended cognition. We do claim, however, that such research is scarce, is typically not about “extended cognition” per se but is readily interpretable as being about extended cognition post hoc (e.g., Auvray et al., 2009), and can be more properly understood as not being about extended cognition but other forms of cognition, such as distributed cognition (e.g., Richardson et al., 2007; cf. Amon & Favela, 2019, which describes empirically measurable differences between extended and distributed forms of cognition).

3 Note that we utilize the term “SSDs” broadly to include simple tools with no moving parts—such as canes—and more mechanical instruments—such as Bach‐y‐Rita et al.’s tactile‐vision sensory substitution system (Bach‐y‐Rita, Collins, Saunders, White, & Scadden, 1969). We could make a distinction and refer to the rod used in the current research as a “tool” and the Enactive Torch as a “device.” Moreover, we acknowledge there is controversy concerning when to categorize something (e.g., Braille, canes, cochlear implants, etc.) as “sensory substitution” as opposed to, for example, sensory augmentation, sensory replacement, and so forth (e.g., Auvray & Myin, 2009; Maidenbaum, Abboud, & Amedi, 2014; Wright & Ward, 2019). Though meaningful issues, we do not address them in the current work and aim for a broad and uncontroversial use of “SSD.”

4 Consistent with the notion that *p*‐values and effect sizes should be interpreted as a continuous variable, our interpretations of confidence in rejecting the null hypothesis also lie along a continuum (e.g., Andrade, 2019). Thus, we opt to acknowledge marginal effects in our results.

5 As reported in the summary statistics section, the average *H* minima are calculated based on the average *H* minima of each participant, such that the Enactive Torch condition exhibits a lower *H* minima, compared to the rod condition. Similarly, because the mixed‐effects models calculate effects by taking into account the slopes of individual participants, versus pooling all of the trials, the findings indicate that the Enactive Torch condition has lower *H* minima. However, averaged across individual trials (versus each individual participant), the direction of the Enactive Torch condition exhibited a higher average *H* minima, compared to the rod condition. We note this discrepancy for researchers who perform follow‐up analyses with our publicly available dataset.

6 We thank a reviewer for suggesting we make clear the connections that our claim of “functional equivalency” has with other concepts in the relevant literature. For example, defining extended cognition in terms of functional equivalency is comparable with the “parity principle” argument for extended mind (Clark & Chalmers, 1998). In short, the parity principle states that if an object outside the body (e.g., iPhone) plays the same functional role as processes in the brain do (e.g., hippocampal neurons), then those external objects should count as cognitive. Another concept, “complementarity,” has also been appealed to in the extended cognition literature (Sprevak, 2019). With complementarity, an object does not have to be functionally similar to a biological process but can become an extended part of the cognitive system when it is tightly integrated somewhere in the brain‐body‐environment system.

7 We thank a reviewer for suggesting this specific line of future research.
